# Treatment resistant manic episode with commorbidity with borderline personality disorder (BPD)

**DOI:** 10.1192/j.eurpsy.2021.1662

**Published:** 2021-08-13

**Authors:** A. Sarafopoulos, D. Antoniadis, V. Karpouza

**Affiliations:** 1 4th Picu, Psychiatric Hospital of Thessaliniki, Thessaloniki, Greece; 2 4th Picu, Psychiatric Hospital of Thessaloniki, Thessaloniki, Greece

**Keywords:** manía, BPD

## Abstract

**Introduction:**

A 47 y.o. female patient was admitted to the female PICU with a manic episode. Upon admission she presented with mood elation, psychosis, pressured speech, lack of sleep and agitation.

**Objectives:**

To investigate negative prognostic factors such as the co-morbidity with a personality disorder in patients presenting with severe mania.

**Methods:**

The patient was assessed regularly by the psychiatric team consisting of a CT doctor and one General Adult Consultant. Appropriate psychological assessments for mania and laboratory investigations took place.

**Results:**

The patient initially scored above 45 in the Young Mania Rating Scale (YMRS), establishing a diagnosis of severe mania. She was treated with Olanzapine titrated up to 20mg OD and augmentation with Lithium treatment. Lithium plasma levels were at 0,6. Due to the treatment resistant manic presentation a second antipsychotic agent was administered and the patient was also treated with Zuclopenthixol depot 400mg every two weeks. Clinical improvement was observed after 16 days from admission.

**Conclusions:**

The clinical team wondered about the clinical challenges of the case. According to the literature having a Personality Disorder diagnosis is a negative prognostic factor for patients with mania and this is relevant to this case as well.

**Disclosure:**

No significant relationships.

